# Identification of a Shared Genetic Susceptibility Locus for Coronary Heart Disease and Periodontitis

**DOI:** 10.1371/journal.pgen.1000378

**Published:** 2009-02-13

**Authors:** Arne S. Schaefer, Gesa M. Richter, Birte Groessner-Schreiber, Barbara Noack, Michael Nothnagel, Nour-Eddine El Mokhtari, Bruno G. Loos, Søren Jepsen, Stefan Schreiber

**Affiliations:** 1 Institute for Clinical Molecular Biology, University Medical Center Schleswig-Holstein, Kiel, Germany; 2 Department of Operative Dentistry and Periodontology, University Medical Center Schleswig-Holstein, Kiel, Germany; 3 University Medical Center Carl Gustav Carus der Technischen Universität Dresden, Zentrum für Zahn-, Mund- und Kieferheilkunde, Poliklinik für Zahnerhaltung, Dresden, Germany; 4 Institute of Medical Informatics and Statistics, University Medical Center Schleswig-Holstein, Kiel, Germany; 5 Clinic of Cardiology, University Medical Center Schleswig-Holstein, Kiel, Germany; 6 Department of Periodontology, Academic Centre for Dentistry Amsterdam (ACTA), VU University Amsterdam, Amsterdam, The Netherlands; 7 Department of Periodontology, Operative and Preventive Dentistry, University of Bonn, Bonn, Germany; University of Oxford, United Kingdom

## Abstract

Recent studies indicate a mutual epidemiological relationship between coronary heart disease (CHD) and periodontitis. Both diseases are associated with similar risk factors and are characterized by a chronic inflammatory process. In a candidate-gene association study, we identify an association of a genetic susceptibility locus shared by both diseases. We confirm the known association of two neighboring linkage disequilibrium regions on human chromosome *9p21.3* with CHD and show the additional strong association of these loci with the risk of aggressive periodontitis. For the lead SNP of the main associated linkage disequilibrium region, rs1333048, the odds ratio of the autosomal-recessive mode of inheritance is 1.99 (95% confidence interval 1.33–2.94; *P* = 6.9×10^−4^) for generalized aggressive periodontitis, and 1.72 (1.06–2.76; *P* = 2.6×10^−2^) for localized aggressive periodontitis. The two associated linkage disequilibrium regions map to the sequence of the large antisense noncoding RNA *ANRIL*, which partly overlaps regulatory and coding sequences of *CDKN2A/CDKN2B*. A closely located diabetes-associated variant was independent of the CHD and periodontitis risk haplotypes. Our study demonstrates that CHD and periodontitis are genetically related by at least one susceptibility locus, which is possibly involved in *ANRIL* activity and independent of diabetes associated risk variants within this region. Elucidation of the interplay of *ANRIL* transcript variants and their involvement in increased susceptibility to the interactive diseases CHD and periodontitis promises new insight into the underlying shared pathogenic mechanisms of these complex common diseases.

## Author Summary

Coronary heart disease (CHD) and periodontitis are the most widespread diseases in the Western industrialized world and pose a substantial health threat to populations worldwide. CHD is a leading cause for premature death, and periodontitis is the major cause for tooth loss in adults over 40 years. Both diseases are associated with similar risk factors such as smoking, diabetes, and gender, and both diseases are further characterized by a chronic inflammatory process. In the last year, several genome studies have identified a region of the human genome near the *CDKN2A* and *CDKN2B* genes as having an influence on CHD. We show that this genetic region, being the most important susceptibility locus for CHD to date, is also associated with a substantial risk increase of aggressive periodontitis. The associated genetic region maps to a genomic region that codes for an “antisense RNA,” which partly overlaps regulatory and coding sequences of genes *CDKN2A/CDKN2B*. The interplay between these common inflammatory complex diseases could be partially due to the shared genetic risk variants of this antisense RNA.

## Introduction

Coronary Heart Disease (CHD) is the leading cause of death worldwide [1],[2]. It is a systemic disease which is propagated by several environmental and behavioural risk factors [3]–[7] with inflammation as an important additional risk [8]. It has a strong genetic basis [9], but despite decades of intensive genetic research, until recently, the sequence variants that confer cardiovascular risk had remained largely unknown. Periodontitis is a complex chronic inflammatory disease, resulting in a loss of connective tissue and bone support of the teeth [10]. It is the major cause of tooth loss in adults above 40 years, and according to the WHO, affects human populations worldwide at prevalence rates of up to 10 to 20% for the most severe forms [11]. Moreover, it is associated with an increase in premature death in adults [12]. Formal genetic studies have demonstrated the genetic basis of periodontitis and indicated that about half of the population variance in chronic periodontitis can be attributed to genetic factors, with a concordance rate of 0.23–0.38 for monozygotic twins [13], and a heritability of 50% for dizygotic twins [14]. But hitherto, association studies with periodontitis have led to controversial results and its inherited components have remained unexplained [15].

Epidemiological studies demonstrated an association between the presence of CHD and periodontitis [16],[17], which is dependent on the severity of periodontal disease [18],[19]. CHD is also propagated by risk factors similar to periodontitis. Both diseases share smoking as a major environmental risk factor and relate to diabetes mellitus, obesity, and gender [6], [7], [20]–[22]. Recent studies also demonstrated similarities in the spectrum of bacteria in the oral cavity and in coronary plaques [23],[24], and both diseases are characterized by an imbalanced immune reaction and a chronic inflammatory process [25],[26]. Periodontitis is also associated with elevated C-reactive protein levels [27], a phenotype for which common genetic risk factors for periodontitis and atherosclerosis have been discussed [28]. These findings indicate a possible mutual genetic basis underlying both diseases.

**Figure 1:**
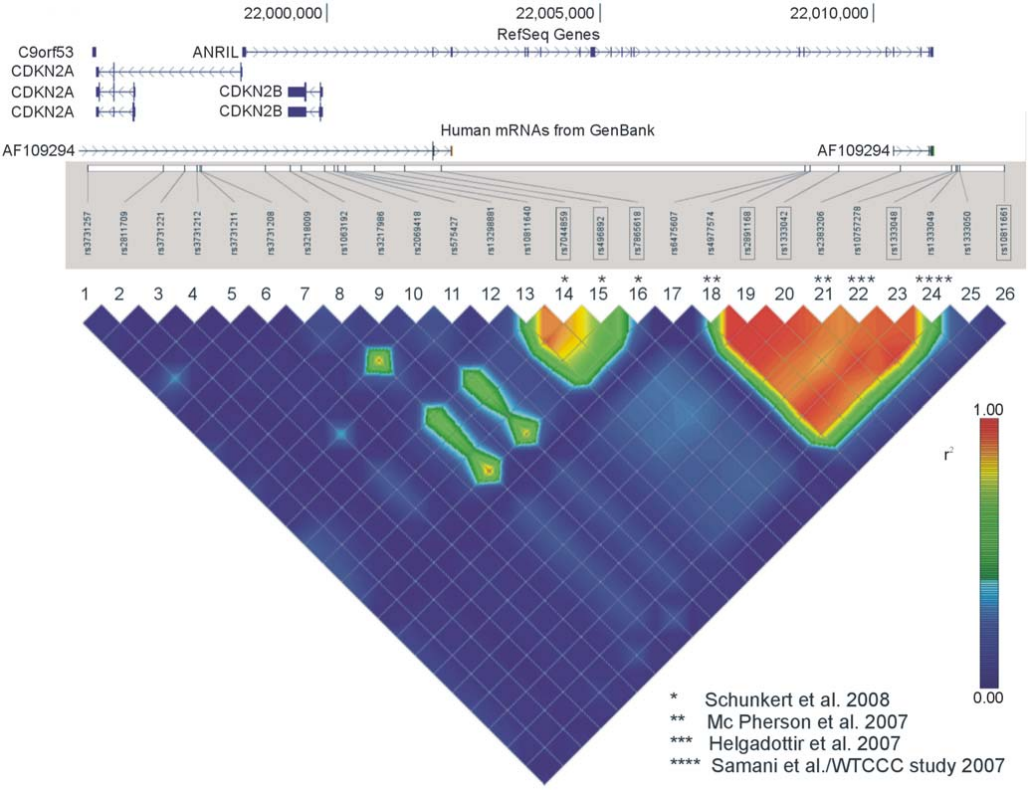
LD Structure (*r^2^*) of the Chromosome 9p21.3 Locus in HapMap CEU. The tagging SNPs of the *CDKN2A/2B* genes (1–13) are given to indicate the extent of these genes on the LD map. They are the only known genes within this genetic region (NCBI build36). Further SNPs are placed on the LD map: the three tagging SNPs of the adjacent AgP/CHD associated LD region (14–16), the four GWAS lead-SNPs of the main AgP/CHD associated LD region (18, 21, 22, 24), the three candidate SNPs selected for this study (19, 20, 23), and the T2D associated SNP (26). SNPs which were genotyped in this study are underlined, the four CHD-associated GWAS lead SNPs and the three CHD associated SNPs of the second LD region are marked by asterisks. SNP 17 and 25 are included into the figure to describe the distinct borders of the LD region. They are located immediately 5′ and 3′ to rs4977574 and rs1333049, respectively). All RefSeq genes and transcripts of this region are placed on the physical gene map, which has been aligned to the LD map and SNP positions below.

Recently, four independent genome-wide association studies (GWAS) reported a strong association of a region of elevated linkage disequilibrium (LD) on human chromosome *9p21.3*, located upstream of the *CDKN2A* and *CDKN2B* genes [29]–[32]. The first two studies identified three individual SNPs (rs238206 [29], *P* = 6.7×10^−6^; rs10757274 [29], *P* = 3.7×10^−6^, and rs10757278 [30], *P* = 1.5×10^−7^), which were in high LD in HapMap [33] CEU (*r^2^*≥0.86). Two subsequent GWA studies [31],[32] identified an association of rs1333049 (*P* = 1.79×10^−14^ and *P* = 3.4×10^−6^, respectively) which is in strong LD with rs10757278 in HapMap CEU and which is flanking the proximate border of the LD region (Figure 1). A subsequent meta-analysis in seven different populations confirmed the association of this region with CHD [34], making it the best replicated genetic risk locus of CHD to date. To investigate whether this locus may also relate to the risk for periodontitis, we investigated a possible association of this LD region with aggressive periodontitis (AgP), the most extreme form of periodontal diseases for which it is assumed that genetic factors play a greater role in the susceptibility than for chronic periodontitis [15].

## Results

In the first stage of the experiment, we selected three SNPs of this LD region (rs2891168, rs1333042 and rs1333048), and verified the putative associations of these SNPs with CHD in a large population of 1,104 early-onset CHD patients (age at disease onset <55 years) and 736 healthy ethnically matched controls (Table 1). All SNPs gave evidence for association with CHD (Table 2, for genotypes refer to Table S1), with SNP rs2891168 being the marginally most significant (*P_multiplicative_* = 1.1×10^−6^, OR = 1.42 [95% confidence interval 1.23–1.64]). We subsequently replicated the association signals in a second independent sample set that comprised the same numbers of CHD patients (age of disease onset <65 years) and of healthy ethnically matched controls as the first sample set. Again, all genotyped SNPs gave similar evidence for association with CHD, *P_multiplicative_* = 2.5×10^−5^ (OR of 1.33 [95% confidence interval 1.16–1.51]) for the most significant, SNP rs1333042 (Table 2).

**Table 1:** Baseline Characteristics of the Study Populations.

	Verification		Replication		Verification		Replication	
	CHD cases	Controls	CHD cases	Controls	Generalized AgP	Controls	Localized AgP	Controls
**Subject characteristic**								
Individuals, n	1,097	736	1,104	736	151	736	137	368
Men (%)	918 (83.7)	222 (30.2)	829 (75.1)	366 (49.7)	53 (35.1)	385 (52.3)	54 (39.4)	184 (50.0)
Women (%)	179 (16.3)	510 (69.3)	275 (24.9)	370 (50.3)	96 (63.6)	351 (47.7)	83 (61)	184 (50.0)
Mean age (SD) at first diagnosis	48 (5.7)	-	57 (6.4)	-	30 (5.6)	-	29 (5.1)	-
Mean (SD) age at examination	57 (5.9)	42 (12.7)	65 (6.1)	46 (13.1)	38 (6.6)	63 (7.4)	37 (7.0)	37 (9.6)
**Study information**								
Recruitment period	2003–05	2003–06	2003–05	2003–06	2004–06	2005–06	2004–06	2005–06
**Phenotype, n (%)**								
Affected teeth, n	-	-	-	-	13 (5.8)	-	4 (1.4)	-
Myocardial Infarction	727 (66.3)	-	655 (59.3)	-	0	32 (4.4)	0	1 (0.3)
Family history CHD	537 (48.6)	-	423 (38.3)	-	No report	113 (15.4)	No report	40 (10.9)
**AgP risk factor, n (%)**								
Ever smoked	868 (79.1)	-	755 (68.4)	-	83 (55.0)	382 (51.9)	59 (43.1)	200 (54.4)
Diabetes mellitus	195 (17.8)	-	218 (19.8)	-	2 (1.3)	48 (6.5)	1 (0.7)	0

Values are given as mean (standard deviation [SD]) when appropriate). Affection status was based on reported history (except for the number of affected teeth which was analyzed on the basis of radiographs by the clinicians, smoking was estimated by self-report). CHD – Coroanry Heart Disease, AgP – Aggressive Periodontitis.

**Table 2:** Candidate SNP Associations of the Main CHD Associated LD Region in Coronary Heart Disease.

CHD panels	SNP	Genotypic					Recessive			Multiplicative			Dominant		
		*P*	OR het	CI 95%	OR hom	CI 95%	*P*	OR	CI 95%	*P*	OR	CI 95%	*P*	OR	CI 95%
**Verification**	**rs2891168**	5.8×10^−6^	1.44	1.13–1.84	2.02	1.52–2.68	8.6×10^−5^	1.61	1.27–2.04	1.1×10^−6^	1.42	1.23–1.64	3.5×10^−5^	1.62	1.29–2.04
	**rs1333042**	5.8×10^−6^	1.49	1.16–1.91	2.02	1.52–2.68	1.5×10^−4^	1.56	1.24–1.97	1.2×10^−6^	1.42	1.23–1.64	2.0×10^−5^	1.66	1.32–2.10
	**rs1333048**	3.9×10^−5^	1.36	1.06–1.75	1.92	1.44–2.55	1.6×10^−4^	1.57	1.24–1.98	7.6×10^−6^	1.39	1.20–1.60	3.3×10^−4^	1.54	1.22–1.95
**Replication**	**rs2891168**	3.4×10^−4^	1.36	1.09–1.69	1.69	1.30–2.21	3.4×10^−3^	1.40	1.12–1.76	7.6×10^−5^	1.30	1.14–1.49	3.8×10^−4^	1.30	1.14–1.49
	**rs1333042**	7.3×10^−5^	1.46	1.17–1.83	1.74	1.34–2.27	5.2×10^−3^	1.37	1.10–1.71	2.5×10^−5^	1.33	1.16–1.51	3.8×10^−5^	1.33	1.16–1.51
	**rs1333048**	2.1×10^−3^	1.31	1.05–1.64	1.59	1.22–2.07	1.1×10^−2^	1.33	1.07–1.67	5.1×10^−4^	1.26	1.11–1.44	1.7×10^−3^	1.26	1.11–1.44

Association Statistics are shown for the two case and control panels of Coronary Heart Disease (CHD). Given are the odds ratios (OR), their 95% confidence intervals (CI 95%), and the *P* values which were obtained either from a likelihood-ratio test (genotypic model) or from a Wald test (autosomal-dominant, multiplicative, and recessive models). Values are given after adjustment for the covariates smoking, diabetes, and gender in a logistic regression model. All other markers showed no significance at the 5% test level under either of the models. *P*_Hardy-Weinberg Equilibrium_ in controls was >0.05 for all markers in all experiments performed.

### Association of the Chromosome 9p21.3 Locus with Generalized AgP

After verification of the CHD associations, we genotyped these SNPs in 159 German periodontitis patients with the most extreme phenotype of AgP, generalized AgP (≥50% bone loss at ≥7 teeth below the age of 35), and 736 independent ethnically matched healthy controls (Table 1). All SNPs were associated with periodontitis, with a multiplicative OR of 1.42 (95% confidence interval 1.11–1.81), *P* = 4.8×10^−3^ and 1.42 (1.11–1.81), *P* = 5.2×10^−3^ for the two most significant SNPs rs1333042 and rs1333048, respectively (Table S2, Table S3). Next, we adjusted for the established AgP risk factors smoking, type 2 diabetes, and the potential confounder gender. Upon adjustment, the effect of the rare alleles of the analyzed markers remained highly significant and was similar to that seen in the unadjusted analysis (Table 3). We considered a full genotypic model as well as a dominant, a multiplicative, and a recessive model for the rare marker allele in a logistic regression analysis. Recessive and multiplicative models yielded *P*-values of similar magnitude for all three markers, with SNP rs1333048 showing the strongest association under the recessive model (*P* = 6.9×10^−4^, OR = 1.99 [95% confidence interval 1.33–2.94]). In line with this, genotypic ORs of SNP rs1333048 equalled 1.12 (0.70–1.80) for heterozygotes and 2.13 (1.31–3.50) for homozygotes, suggesting an underlying genetic model somewhere between the recessive and the multiplicative one. This is supported by the Akaike's Information Criterion (AIC; Table S4), which do not differ between the recessive and the multiplicative model by more than 3.0 for any of the SNPs.

**Table 3:** Candidate SNP Associations of the Main CHD Associated LD Region in Aggressive Periodontitis.

AgP panels	SNP	Genotypic					Recessive			Multiplicative			Dominant		
		*P*	OR (het)	CI 95%	OR (hom)	CI 95%	*P*	OR	CI 95%	*P*	OR	CI 95%	*P*	OR	CI 95%
**Gen.**	**rs2891168**	4.9×10^−3^	1.06	0.68–1.69	1.05	1.26–3.34	8.8×10^−4^	1.97	1.32–2.93	4.4×10^−3^	1.44	1.12–1.86	1.4×10^−1^	1.36	0.91–2.09
	**rs1333042**	7.7×10^−3^	1.10	0.70–1.77	2.00	1.24–3.28	1.6×10^−3^	1.89	1.27–2.79	4.8×10^−3^	1.44	1.12–1.85	1.2×10^−1^	1.40	0.93–2.16
	**rs1333048**	3.6×10^−3^	1.12	0.70–1.80	2.13	1.31–3.50	6.9×10^−4^	1.99	1.33–2.94	2.5×10^−3^	1.48	1.15–1.92	9.6×10^−2^	1.44	0.95–2.23
**Local.**	**rs2891168**	4.6×10^−2^	1.40	0.86–2.34	2.09	1.17–3.77	3.4×10^−2^	1.68	1.03–2.71	1.4×10^−2^	1.45	1.08–1.94	5.5×10^−2^	1.59	1.00–2.58
	**rs1333042**	7.1×10^−2^	1.44	0.87–2.42	1.97	1.10–3.54	6.7×10^−2^	1.56	0.96–2.48	2.2×10^−2^	1.40	1.05–1.88	5.6×10^−2^	1.59	1.00–2.61
	**rs1333048**	2.1×10^−2^	1.56	0.94–2.64	2.30	1.28–4.20	2.6×10^−2^	1.72	1.06–2.76	5.9×10^−3^	1.52	1.13–2.05	2.4×10^−2^	1.76	1.09–2.91

Association Statistics are shown for the two case and control panels of Generalized Aggressive Periodontitis (Gen.) and Localized Aggressive Periodontitis (Local.). Given are the odds ratios (OR), their 95% confidence intervals (CI 95%), and the *P* values which were obtained either from a likelihood-ratio test (genotypic model) or from a Wald test (autosomal-dominant, multiplicative, and recessive models). Values are given after adjustment for the covariates smoking, diabetes, and gender in a logistic regression model. All other markers showed no significance at the 5% test level under either of the models.

### Association of the Chromosome 9p21.3 Locus with Localized AgP

We replicated the associations in an independent population of 146 German periodontitis patients with the less severe AgP phenotype localized AgP (≥50% bone loss at 2–6 teeth below the age of 35), and further 368 ethnically matched healthy controls. All SNPs were found to be significantly associated at both the multiplicative and the genotypic level. SNP rs1333048 was again most significant, with a recessive OR of 1.70 (95% confidence interval 1.06–2.68), *P* = 2.5×10^−2^ (Table S2). After adjustment for the covariates smoking, type 2 diabetes and gender, all SNPs remained significant in the multiplicative genetic model, but not so under the recessive model. The smallest *P*-value was again observed for SNP rs1333048 (*P_multiplicative_* = 5.9×10^−3^) (Table 3). However, genotypic ORs were similar, albeit slightly higher than those observed for generalized AgP (OR_het_ = 1.56 [0.94–2.64], OR_hom_ = 2.30 [1.28–4.20]), and AIC values between the two models differed by less than 3.0 (Table S4), again suggesting a not completely autosomal-recessive model for the genetic effect of this SNP.

The minor allele of rs1333048 has a frequency of 54% in the combined AgP case samples and of 45% in the combined controls (Table S3). These frequencies correspond to those found in this LD region by the WTCCC study (55% in cases, 47% in controls) [31] and by the German myocard infarct study (54% in cases, 48% in controls, [32]).

### Association Analysis of the Proximal CHD Associated LD Region

The analyzed LD region is defined by a region of moderate LD (average D′ approximately 0.60) stretching at the 5′ site [34] (Figure 1). There, a second region of elevated LD, moderately in LD with the main CHD-associated LD region, harbours further CHD associated SNPs [32],[34] (Figure 1). Three tagging SNPs (rs7044859, rs1292136 [recently renamed rs496892], and rs7865618) were identified to be sufficient to characterize the main haplotypes of this region [34]. To test these SNPs for possible associations with AgP, we performed a two step case-control analysis. If a shared genetic cause for both diseases is assumed, association with the primary causal polymorphism will be consistent in clinical cohorts of related diseases, while SNP associations secondary to the main associated LD structure are much less likely to be consistently observed, due to different LD patterns in different cohorts. As with the GWA studies on CHD, in our study, the 3′ LD region gave stronger association signals than the second LD region. Among the three tagging SNPs, only SNP rs496892 was significant in both AgP populations after adjustment. Here, the OR was 1.36 (95% confidence interval 1.04–1.79), *P* = 2.4×10^−2^ for generalized AgP, and 1.38 (95% confidence interval 1.02–1.86), *P* = 3.6×10^−2^ for localized AgP (Table 4, Table S3) in the multiplicative model.

**Table 4:** Candidate SNP Associations of the Second CHD Associated LD Region and the T2D Associated SNP.

AgP panels	SNP	Genotypic					Recessive			Multiplicative			Dominant		
		*P*	OR (het)	CI 95%	OR (hom)	CI 95%	*P*	OR	CI 95%	*P*	OR	CI 95%	*P*	OR	CI 95%
**Gen.**	**rs7044859**	2.1×10^−2^	0.77	0.49–1.23	0.48	0.28–0.83	1.4×10^−2^	0.58	0.38–0.89	6.5×10^−2^	0.69	0.53–0.90	5.5×10^−2^	0.65	0.42–1.02
	**rs496892**	5.3×10^−2^	1.63	1.00–2.73	1.91	1.10–3.38	1.5×10^−1^	1.36	0.89–2.05	2.4×10^−2^	1.36	1.04–1.79	2.6×10^−2^	1.72	1.08–2.82
	**rs7865618**	1.8×10^−1^	0.74	0.49–1.12	0.64	0.38–1.07	2.6×10^−1^	0.76	0.47–1.20	7.5×10^−2^	0.79	0.61–1.02	7.7×10^−2^	0.71	0.49–1.04
	**rs10811661**	5.9×10^−1^	0.59	0.22–1.79	0.68	0.26–1.99	7.2×10^−1^	1.08	0.71–1.66	9.8×10^−1^	1.01	0.71–1.45	4.0×10^−1^	0.66	0.25–1.90
**Local.**	**rs7044859**	5.6×10^−1^	1.02	0.60–1.80	0.80	0.45–1.46	2.9×10^−1^	0.79	0.50–1.22	3.9×10^−1^	0.88	0.66–1.17	7.7×10^−1^	0.93	0.56–1.57
	**rs496892**	9.9×10^−2^	1.51	0.89–2.64	1.92	1.05–3.55	1.3×10^−1^	1.44	0.90–2.28	3.6×10^−2^	1.38	1.02–1.86	6.0×10^−2^	1.64	0.99–2.79
	**rs7865618**	6.2×10^−2^	0.64	0.40–1.02	0.53	0.29–0.95	1.6×10^−1^	0.69	0.40–1.15	2.4×10^−2^	0.72	0.53–0.95	2.2×10^−2^	0.61	0.39–0.93
	**rs10811661**	2.4×10^−2^	n.a.		n.a.		1.1×10^−1^	0.69	0.43–1.09	2.7×10^−1^	0.79	0.52–1.21	9.8×10^−1^	n.a.	

Associations Statistics of the three SNPs in Aggressive Periodontitis which tag the second CHD associated LD region and of the SNP which is associated with T2D, shown for the two case and control panels of Generalized Aggressive Periodontitis (Gen.) and Localized Aggressive Periodontitis (Local.). Given are the odds ratios (OR), their 95% confidence intervals (CI 95%), and the *P* values which were obtained either from a likelihood-ratio test (genotypic model) or from a Wald test (autosomal-dominant, multiplicative, and recessive models). Values are given after adjustment for the covariates smoking, diabetes, and gender in a logistic regression model. All other markers showed no significance at the 5% test level under either of the models.

Statistical evidence of the CHD association signals suggested that the main associated LD region alone did not fully explain the association with CHD [34]. To test this, we performed a haplotype analysis [35] and pooled the two AgP populations to increase statistical power. The analysis indicated that rs1333048 accounted for most of the association with periodontitis (data not shown), similar to the genetic effect observed for the same haplotypic backgrounds in the CHD populations [34].

### Association Analysis with T2D Associated SNP rs10811661

The analyzed LD region is further defined by a recombination hot spot 3′ to rs1333049. (Figure 1). To our knowledge, there is no evidence in the literature for an association with CHD distal to rs1333049 [29]–[32],[34],[36]. Three studies demonstrated an association of rs10811661 with type 2 diabetes [37]–[39] (T2D; 8.6 kb downstream of rs1333049; Figure 1). However, no association of this variant was observed with coronary heart disease [36].

Because T2D is an established risk factor for periodontitis we tested the T2D associated SNP rs10811661 for an additional association with AgP. No association with neither AgP status, prior or after adjustment for smoking, diabetes and gender was detected (Table 4).

## Discussion

In this candidate gene association study we provide evidence for a shared association of the CHD high-risk locus on chromosome 9p21.3 with AgP. We selected SNPs which were located on the same LD region as the lead SNPs of previous GWA studies on CHD, by which they were flanked. We verified the selected SNPs for their association with CHD prior to the replication in the AgP populations. For the initial verification panel we used CHD cases with an age of disease onset <55 years, a particular young age for disease onset, for which it is expected that genetic factors make a particularly high contribution to disease development. The homozygote OR for the three analyzed SNPs ranged from 1.92 to 2.02 and were, thus, in a range similar to the homozygote ORs observed in the WTCCC CHD study (OR = 2.07). The replication panel of CHD patients was on average nine years older than the verification panel. Patient collections of older age at disease onset are generally considered to have a higher proportion of individuals who were more strongly exposed to environmental and/or behavioral risk factors than to genetic ones. In our study, the decreased homozygote ORs in the replication panel, which ranged from 1.59 to 1.74 for the three analyzed SNPs, could be due to this effect, although a simple overestimation of the effect size in the screening panel is an equally likely cause. The controls were ethnically matched with the CHD cases but younger in age. This problem was also encountered earlier by the WTCCC CHD study (cases <66 years vs. two controls panels of 49 years and an age range of 18 to 69 years, respectively). As with the WTCCC study, we also could not adjust for the CHD specific risk factors as the necessary information was not available from the blood donors used as controls. However, the CHD association of this risk LD region appeared to be remarkably uniform in other replication studies and showed no evidence of gene-environmental interactions in that the associations did not appear to be modified by the common CHD risk factors: age gender, smoking history, hypertension, diabetes, obesity, or variations in LDL-, and HDL-cholesterol levels [40].

After the verification of the association of the selected SNPs with CHD, we showed that these variants were also associated with AgP in two independent populations. The robustness of these significant findings was supported by the fact that the inclusion of covariates such as smoking and gender did not notably alter *P*-values or effect sizes. In the explorative analysis we used a population with the most severe phenotype of periodontitis, generalized AgP. Similar to the early-onset CHD phenotype, this most severe form is suspected to be determined by genetic risk factors in particular. In this case panel, we matched the controls for an age older than the general age of onset of mild forms of periodontitis (∼60 years). We considered this matching as appropriate to minimize a possible stratification by individuals with undiagnosed periodontal diseases because less severe forms of periodontitis are not easily diagnosed at a young age and the information of the periodontal status of the controls was self-reported. Because the locus on chromosome 9p21.3 is associated with CHD, it could have an effect on survival. Hence, older controls may represent “survivors” and do not comprise the complete gene pool of the population. This could cause stratification. To assess this problem, we matched the controls in the replication panel for the age of the AgP cases to better mirror the gene pool of the average population. The independence of the association signals from the age of the controls in the replication further confirmed the robustness of the shared association of this locus in CHD and AgP. This robustness of the findings which indicate a shared susceptibility locus for CHD and AgP was also reflected in the similar frequency difference of the rare alleles in both the WTCCC study on CHD and the presented study on AgP, which both differed by approximately 10% between cases and controls. The observed association of a shared susceptibility locus for AgP and CHD suggests a shared genetic cause for these diseases which implies a partly overlapping genetic mechanism of disease development. However, it is likely that by hitherto unknown mechanisms, one disease may also have a direct causal influence on the susceptibility of the other. The mechanisms of these interactions await further elucidation. It can also be argued that some of the epidemiological associations between periodontitis and CHD can be explained by shared socioeconomic risk factors which promote the development of both diseases. If so, AgP cases would be likely to develop future CHD. Likewise, CHD case populations would comprise significant proportions of unphenotyped periodontitis patients. Whereas this might be true for the common forms of chronic periodontitis and late onset CHD, it is unlikely for the severe early onset forms of these diseases. To further limit the potential cause of stratification, we employed cases with a particularly young age and a strong phenotype for both diseases, where the effect of environmental or behavioural factors are limited, and who are considered to carry a strong genetic burden. To add evidence to a genetic rather than a socioeconomical association, the statistical data of the risk LD regions did not appear to be modified by the common environmental and behavioural risk factors known to increase the susceptibility for CHD and/or periodontitis.

Although small compared to other association studies on complex diseases, the presented AgP population represents, to date and to our knowledge, the largest population of this phenotype worldwide. It could be argued that studies with such small sample sizes are prone to increased type I and type II errors. However, for the explorative study we had a statistical power of >90% to detect the observed genetic effects but a confirmation of the described association in further and larger AgP populations would be desirable.

The nearest described protein coding genes within the region of our associated SNPs are the genes *CDKN2A/2B*, which are located >104 kb in 5′ direction and are not in LD with the analyzed haplotypes (*r^2^*<0.15). They encode inhibitors of the genes *CDK4/CDK6* and are believed to play critical roles in cell proliferation and tumor suppression. A third gene, *MTAP*, is located further upstream in close proximity. Many studies also reported *MTAP* function as a tumor suppressor gene and described an inverse correlation between MTAP protein levels and the progression of various tumors [41]–[45]. Interestingly, the association of MTAP activity and interferon sensitivity has been reported [41],[44],[46], indicating a possible role in immune response. Both analyzed risk LD regions do not map to a sequence of these genes, but are embedded in the large antisense noncoding RNA (ncRNA) *ANRIL* [47], belonging to a class of genes thought to be part of the regulatory repertoire of the transcriptome [48]. This gene overlaps *CDKN2B* but does not share any coding nucleotide sequences. Interestingly, mRNA AF109294, which is thought to encode a hypothetical MTAP fusion protein mRNA, also partially overlaps *ANRIL*, sharing two exons. Expression data suggested a coordinated transcriptional regulation of *ANRIL*, *CDKN2A* and *CDKN2B* [47], and expression in tissues involved in atherosclerosis, like vascular endothelial cells, macrophages, and coronary smooth muscle cells, had been shown [40]. The function and mechanism of action for this ncRNA is yet unknown. Full length deletion of *ANRIL* was associated with cutaneous melanoma [47] and the CHD and AgP high-risk haplotype, overlapping exons 13–19 of *ANRIL*, is associated with diseases that also share a phenotype of abnormal cell proliferation. A shared function of the neighbouring genes *CDKN2A/2B* and *MTAP* is the regulation of cell proliferation/tumor suppression. It is tentative to speculate a mechanistic link between the overlapping disease phenotypes of impaired cell proliferation, and a potential transcriptional regulatory role of the ncRNA *ANRIL*. The interplay of these genes in the tissue specific regulation of cell proliferation could be mediated by this antisense RNA. It is intriguing that the CHD and AgP phenotypes share high-risk LD regions, whereas T2D has apparently independent risk variants within this region. Elucidation of the interplay of *ANRIL* transcript variants and their involvement in increased susceptibility to interactive diseases like CHD and periodontitis promises new insight into the underlying partially shared pathogenic mechanisms, and will open up new avenues in the understanding of the development of these complex common diseases.

## Methods

### Study Populations

The CHD patients comprised a population-representative collection of unrelated Germans (Table 1). They were recruited from Schleswig-Holstein, the northernmost region in Germany, through the population-based PopGen biobank [49]. In the recruitment area, all coronary angiograms of any of the five cardiac catheterization laboratories were screened. Study subjects were required to have coronary catheterization demonstrating significant CHD (at least a 70% stenosis in one major epicardial coronary vessel). 1,104 cases had a diagnosed disease onset <55 years, of whom a subset of 596 individuals had suffered a myocardial infarction. The majority (90.3%) had a history of severe CHD and had undergone a coronary revascularization procedure (percutaneous coronary intervention or coronary artery bypass grafting). The controls were obtained from the Blood Service of the University Hospital Schleswig-Holstein. Information about the age and gender was available. Written informed consent was obtained from all participants and the recruitment and the experimental protocols were approved by the institutional ethics review board and data protection authorities.

The AgP patients were recruited from througout Germany. Only patients of German ethnicity were included, determined by the location of both parental birthplaces. Prior to the study, the genetic sub-structure of the German population had been assessed [50], indicating only negligible sub-structures and therefore allowing a joint analysis of all individuals. A further inclusion criterion was age at diagnosis ≤35 years. A set of full-mouth dental radiographs was available for confirmative periodontal bone scoring, ≥2 teeth with ≥50% periodontal bone loss was defined as inclusion criterion. The sub-phenotype of localized AgP was characterized by ≥50% bone loss at 2–6 teeth; the sub-phenotpye generalized AgP was characterized by ≥50% bone loss at ≥7 teeth. The ethnically matched controls used for generalized AgP association study were randomly identified on the basis of the local population registry and the controls used for localized AgP were obtained from the Blood Service of the University Hospital Schleswig-Holstein. All controls underwent an additional physical examination at the PopGen facilities to obtain information on general health status. Information on the oral health status and physical risk factors (e.g. smoking, diabetes) was obtained from questionnaires completed during medical consultation. Additionally, a clinical checkup was subsequently performed. All controls self reported to be free of periodontitis.

### Genotyping

Genomic DNA was extracted from blood samples (Invisorb Blood Universal Kit, Invitek, Berlin, Germany) and amplified by whole genome amplification (GenomiPhi, Amersham, Uppsala, Sweden). Genotyping was performed using the SNPlex and TaqMan GenotypingSystem (Applied Biosystems, Foster City, CA, USA) on an automated platform, employing TECAN Freedom EVO and 96-well and 384-well TEMO liquid handling robots (TECAN, Männedorf, Switzerland). Genotypes were generated by automatic calling using the Genemapper 4.0 software (Applied Biosystems) with the following settings: sigma separation >6, angle separation for 2 cluster SNPs <1.2 radians, median cluster intensity >2.2 logs. Genotypes were additionally reviewed manually and call rates >95% in each sample set were required.

### Statistical Analysis

Power calculations were performed using PS Power and Sample Size Calculations [51]. Markers were tested for deviations from Hardy-Weinberg equilibrium in controls before inclusion in the analysis (http://ihg2.helmholtz-muenchen.de/cgi-bin/hw/hwa2.pl, α = 0.05). Single-marker case-control analysis was performed using Haploview v4.0 [52], PLINK v2.049 [53], and FamHap [35]. LD measures were plotted with the GOLD program [54]. We assessed the significance of associations with or between single-locus genotypes using χ^2^ and Fisher's exact tests for 2×2 and 2×3 contingency tables where applicable. Logistic regression analysis was performed in R v2.7.2 [55]. Significance was assessed by a Wald test and by a likelihood-ratio test.

## Supporting Information

Table S1 Genotype Frequencies for the CHD Cases and Controls.
(0.05 MB DOC)

Table S2 SNP Associations in Aggressive Periodontitis Prior to Adjustment for Covariates.
(0.07 MB DOC)

Table S3 Genotype Frequencies for the AgP Cases and Controls.
(0.06 MB DOC)

Table S4 AIC Values for the Genetic Models Tested in the Study.
(0.06 MB DOC)
