# Supplementary material for: Identification of a Shared Genetic Susceptibility Locus for Coronary Heart Disease and Periodontitis
Source: PLoS Genet. 2009 Feb 13;5(2):e1000378. doi: 10.1371/journal.pgen.1000378 (PMC2632758; doi:10.1371/journal.pgen.1000378)
Supplement: Table S1 — Genotype Frequencies for the CHD Cases and Controls. (0.05 MB DOC) [file pgen.1000378.s001.doc]

**Table S1.** Genotype Frequencies for the CHD Cases and Controls.

1 indicates the major allele, 2 indicates the minor allele of the main CHD associated LD Region.

| CHD |  |  |  |  | **Genotypes cases** |  |  |  | **Genotypes controls** |  |
| --- | --- | --- | --- | --- | --- | --- | --- | --- | --- | --- |
|  | **SNP** | **Allele** |  | **11** | **12** | **22** |  | **11** | **12** | **22** |
|  | **rs2891168** | A/G |  | 187 | 380 | 251 |  | 218 | 308 | 251 |
| **Verification** | **rs1333042** | **A/G** |  | 174 | 380 | 263 |  | 208 | 306 | 156 |
|  | **rs1333048** | **A/C** |  | 176 | 388 | 259 |  | 198 | 321 | 152 |
|  | **rs2891168** | **A/G** |  | 275 | 536 | 285 |  | 240 | 346 | 147 |
| Replication | **rs1333042** | **A/G** |  | 257 | 541 | 298 |  | 236 | 340 | 157 |
|  | **rs1333048** | **A/C** |  | 265 | 537 | 294 |  | 226 | 349 | 158 |
