# Supplementary material for: Identification of a Shared Genetic Susceptibility Locus for Coronary Heart Disease and Periodontitis
Source: PLoS Genet. 2009 Feb 13;5(2):e1000378. doi: 10.1371/journal.pgen.1000378 (PMC2632758; doi:10.1371/journal.pgen.1000378)
Supplement: Table S2 — SNP Associations in Aggressive Periodontitis Prior to Adjustment for Covariates. (0.07 MB DOC) [file pgen.1000378.s002.doc]

|  |  |  |  |  | **Genotypic** |  |  |  |  | **Recessive** |  |  | **Multiplicative** |  |  |  |  | **Dominant** |  |
| --- | --- | --- | --- | --- | --- | --- | --- | --- | --- | --- | --- | --- | --- | --- | --- | --- | --- | --- | --- |
| **AgP panels** | **SNP** |  | ***P*** | **OR (het)** | **CI 95%** | **OR (hom)** | **CI 95%** |  | ***P*** | **OR** | **CI 95%** |  | ***P*** | **OR** | **CI 95%** |  | ***P*** | **OR** | **CI 95%** |
|  | **rs2891168** |  | 7.9 x 10^-3^ | 1.04 | (0.67-1.61) | 1.91 | 1.20-3.06 |  | 1.5 x 10^-3^ | 1.87 | 1.27-2.74 |  | 7.8 x 10^-3^ | 1.39 | 1.09-1.78 |  | 2.0 x 10^-1^ | 1.30 | 0.88-1.95 |
|  | **rs1333042** |  | 8.4 x 10^-3^ | 1.12 | (0.72-1.76) | 1.96 | 1.23-3.15 |  | 1.9 x 10^-3^ | 1.83 | 1.24-2.66 |  | 4.8 x 10^-3^ | 1.42 | 1.11-1.81 |  | 1.1 x 10^-1^ | 1.40 | 0.93-2.11 |
| **Gen.** | **rs1333048** |  | 6.7 x 10^-3^ | 1.07 | (0.69-1.69) | 1.96 | 1.22-3.16 |  | 1.3 x 10^-3^ | 1.87 | 1.27-2.73 |  | 5.2 x 10^-3^ | 1.42 | 1.11-1.82 |  | 1.5 x 10^-1^ | 1.35 | 0.90-2.05 |
|  | **rs7044859** |  | 2.2 x 10^-2^ | 0.834 | (0.54-1.31) | 0.51 | 0.30-0.86 |  | 1.0 x 10^-2^ | 0.58 | 0.38-0.87 |  | 8.6 x 10^-3^ | 0.71 | 0.55-0.92 |  | 1.0 x 10^-1^ | 0.70 | 0.46-1.09 |
|  | **rs496892** |  | 6.3 x 10^-2^ | 1.56 | (0.98-2.56) | 1.84 | 1.08-3.16 |  | 1.5 x 10^-1^ | 1.34 | 0.89-1.99 |  | 2.7 x 10^-2^ | 1.34 | 1.04-1.74 |  | 3.2 x 10^-2^ | 1.65 | 1.06-2.65 |
|  | **rs7865618** |  | 1.4 x 10^-1^ | 0.76 | (0.52-1.13) | 0.61 | 0.36-1.01 |  | 1.6 x 10^-1^ | 0.72 | 0.44-1.12 |  | 4.8 x 10^-2^ | 0.78 | 0.61-1.0 |  | 7.0 x 10^-2^ | 0.71 | 0.50-1.03 |
|  | **rs10811661** |  | 5.1 x 10^-1^ | 0.58 | (0.21-1.72) | 0.69 | 0.27-1.98 |  | 5.8 x 10^-1^ | 1.12 | 0.75-1.70 |  | 8.5 x 10^-1^ | 1.04 | 0.74-1.48 |  | 3.9 x 10^-1^ | 0.65 | 0.28-1.87 |
|  | **rs2891168** |  | 4.6 x 10^-2^ | 1.37 | (0.85-2.24) | 2.03 | 1.16-3.58 |  | 3.2 x 10^-2^ | 1.66 | 1.04-2.64 |  | 1.4 x 10^-2^ | 1.42 | 1.08-1.89 |  | 5.7 x 10^-2^ | 1.55 | 1.00-2.47 |
|  | **rs1333042** |  | 5.4 x 10^-2^ | 1.38 | (0.86-2.28) | 1.98 | 1.14-3.48 |  | 4.0 x 10^-2^ | 1.61 | 1.02-2.53 |  | 1.6 x 10^-2^ | 1.41 | 1.07-1.87 |  | 5.7 x 10^-2^ | 1.56 | 1.0-2.49 |
| **Local.** | **rs1333048** |  | 2.2 x 10^-2^ | 1.51 | (0.93-2.49) | 2.2 | 1.26-3.94 |  | 2.5 x 10^-2^ | 1.70 | 1.06-2.68 |  | 6.3 x 10^-3^ | 1.49 | 1.12-1.98 |  | 2.6 x 10^-2^ | 1.70 | 1.08-2.75 |
|  | **rs7044859** |  | 4.2 x 10^-1^ | 0.92 | (0.55-1.57) | 0.71 | 0.41-1.26 |  | 2.0 x 10^-1^ | 0.76 | 0.49-1.16 |  | 2.1 x 10^-1^ | 0.84 | 0.63-1.11 |  | 4.6 x 10^-1^ | 0.83 | 0.51-1.38 |
|  | **rs496892** |  | 4.5 x 10^-2^ | 1.64 | (0.97-2.83) | 2.07 | 1.15-3.79 |  | 9.2 x 10^-2^ | 1.47 | 0.93-2.30 |  | 1.7 x 10^-2^ | 1.42 | 1.07-1.91 |  | 2.7 x 10^-2^ | 1.77 | 1.08-3.0 |
|  | **rs7865618** |  | 1.4 x 10^-2^ | 0.58 | (0.37-0.90) | 0.49 | 0.27-0.84 |  | 1.1 x 10^-1^ | 0.66 | 0.39-1.09 |  | 6.2 x 10^-3^ | 0.68 | 0.51-0.89 |  | 4.1 x 10^-3^ | 0.55 | 0.36-0.83 |
|  | **rs10811661** |  | 2.4 x 10^-2^ | n.a. |  | n.a. |  |  | 1.9 x 10^-1^ | 0.74 | 0.48-1.16 |  | 4.3 x 10^-1^ | 0.85 | 0.57-1.29 |  | 9.8 x 10^-1^ | n.a. |  |

**Table S2.** SNP Associations in Aggressive Periodontitis Prior to Adjustment for Covariates.

Association Statistics are shown for the two case and control panels of Generalized Aggressive Periodontitis (Gen.) and Localized Aggressive Periodontitis. (Local.). Shown are the values of the three SNPs which tag the main CHD associated LD region, the values of the three SNPs which tag the second CHD associated LD region, and the SNP which is associated with T2D.Given are the odds ratios (OR), their 95% confidence intervals (CI 95%), and the *P* values which were obtained either from a likelihood-ratio test (genotypic model) or from a Wald test (autosomal-dominant, multiplicative, and recessive models). Values are given prior adjustment for the covariates smoking, diabetes, and gender in a logistic regression model.
