# Supplementary material for: Identification of a Shared Genetic Susceptibility Locus for Coronary Heart Disease and Periodontitis
Source: PLoS Genet. 2009 Feb 13;5(2):e1000378. doi: 10.1371/journal.pgen.1000378 (PMC2632758; doi:10.1371/journal.pgen.1000378)
Supplement: Table S3 — Genotype Frequencies for the AgP Cases and Controls. (0.06 MB DOC) [file pgen.1000378.s003.doc]

**Table S3.** Genotype Frequencies for the AgP Cases and Controls.

|  |  |  |  |  | **Genotypes cases** |  |  |  | **Genotypes controls** |  |
| --- | --- | --- | --- | --- | --- | --- | --- | --- | --- | --- |
|  | **SNP** | **Alleles** |  | **11** | **12** | **22** |  | **11** | **12** | **22** |
|  | **rs7044859** | A/T |  | 34 | 81 | 35 |  | 243 | 355 | 128 |
|  | **rs496892** | **A/G** |  | 27 | 77 | 41 |  | 191 | 362 | 164 |
|  | **rs7865618** | **A/G** |  | 58 | 68 | 25 |  | 226 | 343 | 159 |
| Generalized | **rs10811661** | **C/T** |  | 109 | 36 | 6 |  | 340 | 146 | 14 |
|  | **rs2891168** | **A/G** |  | 39 | 63 | 49 |  | 228 | 350 | 150 |
|  | **rs1333042** | **A/G** |  | 36 | 63 | 52 |  | 222 | 342 | 164 |
|  | **rs1333048** | **A/C** |  | 36 | 63 | 51 |  | 218 | 350 | 158 |
|  | **rs7044859** | **A/T** |  | 38 | 65 | 28 |  | 131 | 169 | 67 |
|  | **rs496892** | **A/G** |  | 22 | 68 | 39 |  | 99 | 179 | 81 |
|  | **rs7865618** | **A/G** |  | 55 | 55 | 22 |  | 102 | 177 | 88 |
| **Localized** | **rs10811661** | **C/T** |  | 88 | 47 | 0 |  | 342 | 124 | 16 |
|  | **rs2891168** | **A/G** |  | 32 | 66 | 36 |  | 121 | 179 | 67 |
|  | **rs1333042** | **A/G** |  | 31 | 65 | 38 |  | 118 | 176 | 73 |
|  | **rs1333048** | **A/C** |  | 29 | 67 | 37 |  | 118 | 181 | 68 |

1 indicates the major allele, 2 indicates the minor allele of the main CHD associated LD Region.
