# Supplementary material for: Identification of a Shared Genetic Susceptibility Locus for Coronary Heart Disease and Periodontitis
Source: PLoS Genet. 2009 Feb 13;5(2):e1000378. doi: 10.1371/journal.pgen.1000378 (PMC2632758; doi:10.1371/journal.pgen.1000378)
Supplement: Table S4 — AIC Values for the Genetic Models Tested in the Study. (0.06 MB DOC) [file pgen.1000378.s004.doc]

|  |  | Genoty-pic |  |  |  |  | Reces-sive |  |  |  |  | Multipli-cative |  |  |  |  | Domi-nant |  |  |  |
| --- | --- | --- | --- | --- | --- | --- | --- | --- | --- | --- | --- | --- | --- | --- | --- | --- | --- | --- | --- | --- |
| SNP |  | Gen. AgP | Loc. AgP | CHD #1 | CHD #2 |  | Gen. AgP | Loc. AgP | CHD #1 | CHD #2 |  | Gen. AgP | Loc. AgP | CHD #1 | CHD #2 |  | Gen. AgP | Loc. AgP | CHD #1 | CHD #2 |
| rs2891168 |  | 746.5 | 553.4 | 2031.5 | 2453.0 |  | 744.6 | 553.1 | 2037.9 | 2458.3 |  | 746.9 | 551.4 | 2029.6 | 2451.2 |  | 752.9 | 553.7 | 2036.5 | 2454.4 |
| rs1333042 |  | 747.4 | 554.2 | 2028.8 | 2450.0 |  | 745.6 | 554.2 | 2036.3 | 2459.1 |  | 747.1 | 552.2 | 2026.9 | 2449.1 |  | 752.6 | 553.7 | 2032.6 | 2450.1 |
| rs1333048 |  | 740.4 | 551.8 | 2041.3 | 2456.7 |  | 738.6 | 552.7 | 2045.1 | 2460.4 |  | 740.3 | 549.8 | 2039.3 | 2454.9 |  | 746.8 | 552.1 | 2046.7 | 2457.2 |
| rs7044859 |  | 748.8 | 556.2 | - | - |  | 748.0 | 554.2 | - | - |  | 747.0 | 554.6 | - | - |  | 751.0 | 555.3 | - | - |
| rs496892 |  | 730.8 | 545.5 | - | - |  | 732.7 | 545.8 | - | - |  | 729.5 | 543.7 | - | - |  | 729.3 | 544.4 | - | - |
| rs7865618 |  | 753.8 | 553.9 | - | - |  | 753.8 | 555.5 | - | - |  | 751.9 | 552.3 | - | - |  | 752.1 | 552.4 | - | - |
| rs10811661 |  | 645.4 | 506.5 | - | - |  | 644.3 | 509.4 | - | - |  | 644.4 | 510.7 | - | - |  | 643.8 | 508.1 | - | - |

**Table S4.** AIC Values for the Genetic Models Tested in the Study.

AIC = Akaike's Information Criterion. The mode of inheritance that best explains the underlying associations is the one with the lowest value of the AIC.
